# Dietary restriction for prevention of contrast-induced acute kidney injury in patients undergoing percutaneous coronary angiography: a randomized controlled trial

**DOI:** 10.1038/s41598-020-61895-2

**Published:** 2020-03-23

**Authors:** Franziska Grundmann, Roman-Ulrich Müller, Karla Johanna Ruth Hoyer-Allo, Martin Richard Späth, Eva Passmann, Ingrid Becker, Roman Pfister, Stephan Baldus, Thomas Benzing, Volker Burst

**Affiliations:** 1 Department II of Internal Medicine and Center for Molecular Medicine Cologne, University of Cologne, Faculty of Medicine and University Hospital Cologne, Cologne, Germany; 2 CECAD, University of Cologne, Faculty of Medicine and University Hospital Cologne, Cologne, Germany; 3 Institute of Medical Statistics and Computational Biology, University of Cologne, Cologne, Germany; 4 Department III of Internal Medicine, Heart Center, University Hospital of Cologne, Cologne, Germany; 5 Systems Biology of Ageing Cologne, University of Cologne, Cologne, Germany

**Keywords:** Translational research, Kidney, Acute kidney injury

## Abstract

Short-term dietary restriction (DR) may prevent organ damage from ischemic or toxic insults in animals, but clear evidence in humans is missing. While especially intraarterial administration of contrast media represents a cause of hospital-acquired acute kidney injury (AKI), targeted preventive strategies are not available. This trial investigated the feasibility and effectiveness of pre-interventional DR for preventing AKI in patients undergoing percutaneous coronary intervention (PCI). Patients were randomized to receive a formula diet containing 60% of daily energy requirement (DR group) or ad-libitum food during the 4-day-interval before PCI. Primary endpoint was change of serum creatinine 48 h after PCI (Δcreatinine). Further analyses included incidence of AKI and safety evaluation. Δcreatinine post PCI in the DR group vs. the control group did not show any difference (DR: 0.03(−0.15,0.14)mg/dL vs. control: 0.09(−0.03,0.22)mg/dL;p = 0.797). Subgroup analyses revealed a significant beneficial impact of DR in patients that received ≤100 ml of contrast agent (DR n = 26: Δcreatinine −0.03(−0.20,0.08)mg/dL vs. control n = 24: Δcreatinine 0.10(−0.08,0.24)mg/dL; p = 0.041) and in patients with ≤2 risk factors for AKI (DR: n = 27; Δcreatinine −0.01(−0.18,0.07)mg/dL vs. control n = 31: Δcreatinine 0.09(−0.03,0.16)mg/dl; p = 0.030). Although the primary endpoint was not met, the results of this trial suggest a beneficial impact of DR in low-to-moderate risk patients.

## Introduction

Dietary restriction (DR) is characterized by reduced food intake without inducing poor nutrition and subsumes numerous interventions including general calorie restriction (CR), protein restriction (PR) and various fasting protocols. Short-term DR – usually excercised for several days to weeks– has an extraordinary power to reduce cellular damage susceptibility by promoting resistance towards ischemic insults in rodent models of injury to the liver,^1,2^ kidney^3,4^ brain,^5^ and heart.^6^ Furthermore, DR has also been proven highly effective in preventing or ameliorating chemotoxic injury to the heart (secondary to doxorubicin)^7^ and the kidney (secondary to cisplatinum)^8^ and successfully improved survival in a murine chemotoxic stress model.^9^ Possible mechanisms contributing to this phenomenon are versatile and include improvement of insulin sensitivity and reduction of oxidative stress and free radical induced damage, as well as modification of DNA repair. Alterations in the mechanistic target-of-rapamycin(mTOR)-signaling and other pathways, such as 5′ adenosine monophosphate-activated protein kinase and sirtuin overexpression also contribute to the effect. Whether DR conveys similar beneficial effects in humans is unknown. However, considering the lack of therapeutic options to prevent AKI in the clinical setting, it would provide a powerful strategy to protect organ integrity in those cases in which a critical impact can be anticipated, e.g. prior to scheduled major surgery or before administration of toxic agents.^10^

Due to its functional and structural properties, the kidney is especially sensitive to ischemic as well as toxic attacks, two mechanisms that are central to the pathophysiology of contrast media-induced acute kidney injury (CI-AKI). With an incidence of up to 50% in patients at risk, CI-AKI after percutaneous coronary intervention (PCI) or angiography is a main inducement of hospital-acquired AKI and has been associated with prolonged hospital stay, increased costs, aggravation or initiation of persistent kidney damage, and increased mortality.^11^ Given the exponentially increasing number of angiographic procedures requiring contrast media administration,^12^ the need for strategies to avoid these problems is growing. Various risk factors for CI-AKI have been identified, i.e. old age,^13^ chronic kidney disease,^14^ congestive heart failure,^15^ hypovolemia^16^ and high-volume contrast media^17,18^ administration. Despite a multitude of clinical trials investigating putative protective compounds, to date restoring fluid volume balance still remains the only widely accepted preventive strategy.^19^

Short-term DR has been proven to be feasible and safe in living kidney donors.^20^ Recently, in a first proof-of-principle clinical trial we showed that a 40%-calorie restriction given over 7 days prior to major cardiac surgery – a procedure associated with a high rate of postoperative ischemic AKI – might attenuate impairment of kidney function mirrored by a significantly reduced increase of serum creatinine at 48 h after surgery.^21^ Here, we report the results of a prospective, single-center, randomized, controlled clinical trial that tested the hypothesis that a 4-day diet with a 40% reduction of calorie intake prevents CI-AKI in at-risk-patients undergoing PCI.

## Subjects and Methods

### Study participants

Approval of the study protocol was obtained from the local institutional review board (Ethics committee of the University of Cologne, Cologne, Germany). 80 consecutive adult patients (>18 years) scheduled for elective PCI at the University Hospital of Cologne, who carried at least one of the following risk factors for developing CI-AKI were enrolled: age >70 years, chronic kidney disease with a pre-existing serum creatinine above the normal range (i.e. >1.1 mg/dL in men, >0.9 mg/dL in women), diabetes mellitus, congestive heart failure NYHA 3–4, reduced left ventricular ejection fraction (<50%), or peripheral vascular disease. Exclusion criteria were end-stage renal disease, kidney transplantation, pregnancy and breastfeeding, weight loss of more than 1 kg within 2 weeks prior to enrollment unless due to diuretic medication, body mass index (BMI) <18.5 kg/m^2^ or >35 kg/m^2^, evidence of malignancy or uncontrolled infection, known allergies or intolerance against ingredients of the formula diet used, or the inability to give informed consent. The study was operated in accordance with the Declaration of Helsinki and the good clinical practice guidelines by the International Conference on Harmonization, registered with the German Clinical Trial Register (https://www.drks.de/drks_web/navigate.do?navigationId=trial.HTML&TRIAL_ID = DRKS00004361; Date of registration: 23/04/2013; DRKS-ID: DRKS00004361) and reported to the Federal Institute for Drugs and Medical Devices (BfArM, EudraCT-no: 2012-003696-18), (full study protocol attached in Supplemental document)

### Study design

Between 6 to 4 days prior to the scheduled day of PCI (day 0), potentially qualifying patients were contacted for a screening visit by a member of the study team. After obtaining informed consent anthropometric characteristics were assessed using a bioimpedance scale (Tanita BC-418 Segmental Body Composition Analyzer, Arlington Heights, Illinois 60005, USA). If eligible, patients were randomly allocated in a 1:1 ratio to either receiving a dietary-restricted (DR group) or an ad-libitum diet (control group) using an internet-based computer-randomization system (ALEA Randomisation Service, Amsterdam, NL; provided by the Institute of Medical Statistics and Computational Biology, University of Cologne, Cologne, Germany). Block randomization or stratification was not performed. After randomization, a member of the study team provided a formula diet (Fresubin energy fibre, Fresenius Kabi™, Bad Homburg, Germany) containing 60% of the daily energy expenditure (DEE), as calculated using the Mifflin-St. Jeor equation and individually assessed activity factors to patients in the DR group. All participants in the DR group were instructed not to consume extra food or calorie-containing beverages like alcohol, fruit juices or soft drinks. The diet commenced on pre-interventional day −4 and was maintained until the pre-interventional indicated fasting state. In the control group patients were allowed to ingest food at their own discretion and were encouraged to stick to their normal eating habits, thereby ensuring non-restricted calorie intake. All participants in both groups were provided with diaries and reported their food consumption on a daily basis. In addition, regular phone calls ascertained patients’ wellbeing and served to monitor adherence to the designated protocol. Re-assessment of all participants was performed on day −1, and blood and urine samples were collected on day 0 immediately prior to PCI to obtain pre-PCI baseline data. All patients received volume therapy as standard of care to prevent CI-AKI. Interventional procedures as well as volume therapy were carried out according to the local standard (1 ml/kg body weight per hour for 12 h before and after PCI) and were not influenced by the study protocol. Other putative measures of organ protection, e.g. remote ischemic preconditioning, were not performed throughout the study. Iohexol (Accupaque™ 300, GE Healthcare, Braunschweig, Germany) was used as contrast agent in all patients. Dosing was at the discretion of the treating physician performing the PCI and not influenced by study personnel.

### Outcome parameters

Change in serum creatinine (Δ creatinine) from pre-PCI baseline (0 h) to 48 h after the initiation of PCI was analyzed as primary endpoint. Secondary endpoints were the change of serum creatinine from pre-PCI baseline to 24 h after PCI, the change in urinary Neutrophil gelatinase-associated lipocalin (NGAL) from pre-PCI baseline to 24 h after PCI, the change of cystatin C from pre-PCI baseline to 24 h and 48 h after PCI, the incidence of CI-AKI defined as an increase in serum creatinine of ≥0.5 mg/dL or ≥25% of the pre-PCI baseline value within 48 h after PCI, incidence of AKI as defined by Kidney disease: Improving Global Outcomes (KDIGO) criteria (creatinine parameter only),^22^ mortality, hospitalization and/or need for renal replacement treatment (RRT) within 4 weeks after PCI, and progression of the following biochemical safety parameters from pre-PCI baseline to 24 h and 48 h after PCI: C-reactive protein (CRP), white cell blood count (WBC), creatine kinase (CK), creatine kinase muscle brain type (CK-MB), and lactate dehydrogenase (LDH). Reported changes of laboratory results were calculated as value at specified time point post PCI minus pre-PCI baseline value (day 0).

The decision for initiation of RRT was performed solely by the treating physician and not effected by study personnel.

### Data collection

Baseline demographic characteristics including co-morbidities and medication were assessed after study inclusion. All blood and urine samples at pre-PCI baseline and within 48 h after PCI were obtained at pre-specified time points. Body weight and body composition data were documented at the screening visit and at hospital admittance (day -1). All participants were asked to collect a 24 hour-urine sample at day −2, and calculation of the daily protein intake was carried out by estimating urinary urea nitrogen appearance with the equation proposed by Maroni *et al*.^23^. Patients were encouraged to document any additional food intake on a daily basis. Additionally, the general condition was reported in a 3-point-ordinal scale (rather good, fair, rather not good) as was the sense of hunger (minor, moderate, severe). Duration of PCI, amount of contrast media used and other variables were extracted from the medical records. All patients were followed up until hospital discharge; the occurrence of re-hospitalization, RRT and death was assessed by a telephone call at week 4 after PCI. Documentation of adverse events was performed starting with the first study-related procedure and ending with the last telephone contact at week 4.

### Statistical analysis

A previously published trial^24^ conducted at the Cologne University Hospital observed an increase in serum creatinine 48 h after contrast medium exposure of 0.16 ± 0.24 mg/dl (remote ischemic preconditioning) and 0.47 ± 0.48 mg/dl (no preconditioning), corresponding to a standardized effect size of 0.82. To prove this effect with a power of 80%, a minimum inclusion of 24 patients per group was calculated. To compensate for a 20% effect reduction due to dropouts, 1.56 × 24^25^ patients were included in each group, i.e. after rounding 40 patients.

Primary analysis was performed in an intention-to-treat (ITT) approach and patients were included as randomized. The analysis of the per-protocol group included all randomized patients that were treated according to the protocol (e.g. no delay of surgery), patients in the DR group were included if they had consumed no more than 60% of DEE + 200 kcal within the DR period. Pre specified subgroup analyses were executed for gender, BMI, CI-AKI risk groups, and administered contrast agent volume.

Primary analysis was the treatment comparison by analysis of co-variance (ANCOVA) in the ITT population with covariate serum creatinine at day 0. Missing values were replaced with a last-observation-carried-forward approach. Secondary serum creatinine outcomes were also evaluated by ANCOVA. Other quantitative outcomes were analyzed by Mann-Whitney-U tests, qualitative outcomes by Chi-square or exact Fisher tests. Missing values were not replaced in the secondary endpoint analyses as well as in the per-protocol analysis.

No race/ethnicity-specific analyses were performed, since all included patients were caucasian. Data are reported as means (± standard deviation, SD), medians (interquartile range, IQR), or numbers [n (% of total)]. All statistical testing was two-sided and a p-value < 0.05 was considered significant. Analysis software was SAS 9.3, SAS Institute Inc., Cary, USA and IBM SPSS Statistics Version 23.

## Results

**Figure 1:**
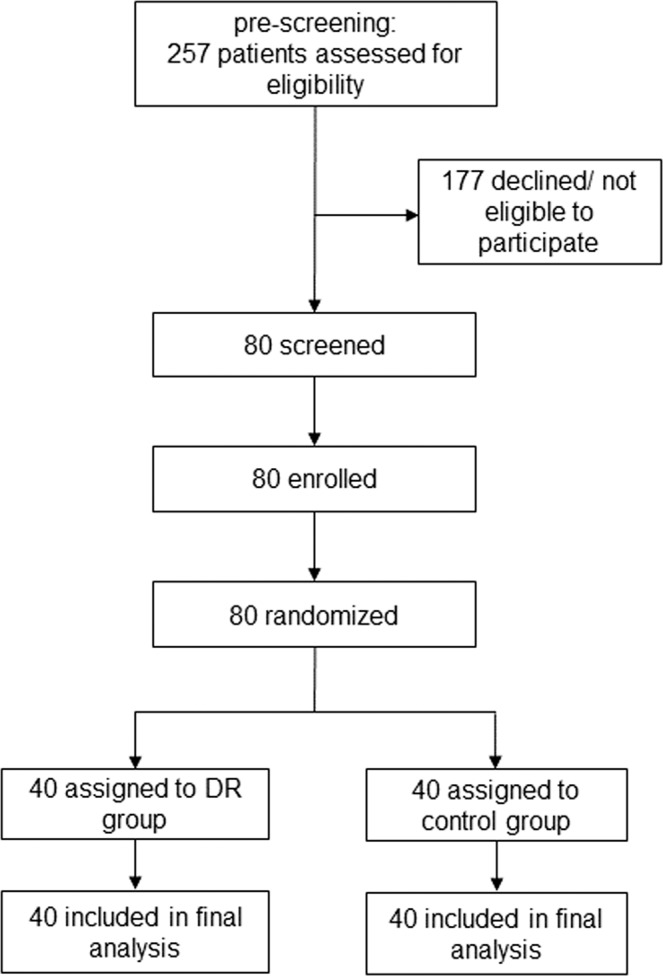
Patient flow and randomization. DR: dietary restriction.

The first patient was included July 10, 2013, the last follow up visit scheduled on October 7, 2016. During this period, 257 potentially eligible patients were identified and contacted. Of these, 177 were found not eligible in pre-screening telephone contact or declined their participation. Eventually, 80 patients were screened, and all of them randomized into the two groups (40 in each group). The recruitment was ended after reaching the enrollment goal as planned. All randomized patients were included in the ITT analysis (Fig. 1). Patient demographics as well as clinical characteristics are shown in Table 1.

**Table 1:** Patient demographics and clinical characteristics.

	DR group (n = 40)	control group (n = 40)	total (n = 80)	p
age [years], median(Q1–Q3)	74.0 (71.5,78.5)	73.5 (70.5,78.0)	74.0 (71.0,78.0)	0.912
male, [n]	28 (70.0%)	31 (77.5%)	59 (73.8%)	0.446
weight at screening [kg], median(Q1–Q3)	81.8 (73.8,92.0)	82.5 (76.8,89.8)	82.5 (76.1,91.4)	0.874
BMI at screening [kg/m^2^], median(Q1–Q3)	28.0 (25.5,31.0)	28.0 (25.0,29.5)	28.0 (25.0,30.5)	0.619
Creatinine at screening [mg/dL], median(Q1–Q3)	1.4 (1.1,1.9)	1.1 (1.0,1.4)	1.3 (1.0,1.5)	0.009
Creatinine day 0 [mg/dL], median(Q1–Q3)	1.3 (1.1,1.7)	1.1 (0.9,1.4)	1.2 (1.0,1.5)	0.004
eGFR (CKD-EPI) [ml/min/1.73m2], median(Q1–Q3)	45 (32,58)	58 (47,71)	51 (42,66)	0.003
Peripheral arterial disease, [n]	2 (5.0%)	5 (12.5%)	7 (8.8%)	0.235
Congestive heart failure, [n]	10 (25.0%)	6 (15.0%)	16 (20.0%)	0.264
Valve disease, [n]	13 (32.5%)	11 (27.5%)	24 (30.0%)	0.626
Coronary artery disease, [n]	26 (65.0%)	31 (77.5%)	57 (71.3%)	0.217
Atrial fibrillation, [n]	12 (30.0%)	11 (27.5%)	23 (28.8%)	0.805
Pulmonary hypertension, [n]	5 (12.5%)	7 (17.5%)	12 (15.0%)	0.531
Hypertension, [n]	35 (87.5%)	26 (65.0%)	61 (76.3%)	0.018
Diabetes mellitus, [n]	13 (32.5%)	12 (30.0%)	25 (31.3%)	0.809
Pace maker, [n]	3 (7.5%)	2 (5.0%)	5 (6.3%)	0.644
AICD, [n]	1 (2.5%)	0 (0.0%)	1 (1.3%)	0.314
Mehran Score	6	5	6	0.200
≤2 risk factors for CI-AKI, [n]	27 (67.5%)	31 (77.5%)	58 (72.5%)	0.317
>2 risk factors for CI-AKI, [n]	13 (32.5%)	9 (22.5%)	22 (55%)	0.317
ACEI / ARB, [n]	32 (80.0%)	31 (77.5%)	63 (78.8%)	0.785
Aldosterone antagonist, [n]	6 (15.0%)	8 (20.0%)	14 (17.5%)	0.556
Beta blocker, [n]	32 (80.0%)	31 (77.5%)	63 (78.8%)	0.785
Calcium antagonist, [n]	12 (30.0%)	8 (20.0%)	20 (25.0%)	0.302
Lipid-lowering drugs, [n]	26 (65.0%)	33 (82.5%)	59 (73.8%)	0.075
Antiplatelet therapy, [n]	30 (75.0%)	35 (87.5%)	65 (81.3%)	0.152
Diuretic, [n]	27 (67.5%)	29 (72.5%)	56 (70.0%)	0.626

DR: dietary restriction; AICD: Automated Implantable Cardioverter-Defibrillator; ACEI/ARB: angiotensin-converting enzyme inhibitor/angiotensin receptor blocker; CI-AKI: contrast media-induced acute kidney injury; eGFR: estimated glomerular filtration rate; CKD-EPI: Chronic Kidney Disease Epidemiology Collaboration.

According to the patients’ diary recordings the median daily calorie intake was 1212(1070,1404) kcal accounting for 60(59,60)% of the calculated DEE in the DR group and 1597(1440,1876) kcal representing 81(70,95)% of the calculated DEE in the control group which would imply a true between-group calorie reduction of only 20%. However, in contrast to the DR group in which the calculation of additionally consumed calories was presumably precise since patients only added minor amounts of comestibles or countable items (e.g. 1 apple, 3 biscuits, etc.) an accurate assessment of valid calorie uptake in the control group was hindered by incomplete or unspecific reports on consumed food quantities. Furthermore, all patients in the control group indicated that they had not changed their diets and had not carried out a calorie restriction on their own. Consequently, the median weight loss in the control group was −0.7(−1.0,0.1) kg whereas patients in the DR group lost −2.3(−2.9,1.5) kg (p < 0.001). In the DR group the considerable weight change was largely due to a decrease in total body water with −2.1 (−4.1,1.0) kg as compared to the control group with −1.1 (−1.6,0.6) kg; p = 0.002). In between-group difference was also evident in the estimated daily protein intake, based on the urinary urea nitrogen appearance (DR: 57(51,64) g/day vs. control group: 69(52,87) g/day, p < 0.033). Anthropometric and bioimpedance characteristics are summarized in Table 2 and Supplemental Table 1.

**Table 2:** Anthropometric characteristics and biochemical parameters.

	DR group (n = 40)	control group (n = 40)	p
Δ weight: screening to day −1, [kg]	−2.3 (−1.9,2.5)	−0.7 (−1.0,−0.1)	<0.001
Δ body water: screening to day −1, [kg]	−2.1 (−4.1,−1.0)	−1.1 (−1.6,0.6)	0.002
calculated daily energy expenditure (DEE), [kcal]	2178 (1821,2370)	2094 (1893,2286)	0.394
reported calorie intake, [kcal]	1212 (1067,1404)	1597 (1440,1876)	<0.001
reported calorie intake, [% of DEE]	60 (59,60)	81 (70,95)	<0.001
daily protein intake calculated from urinary urea nitrogen appearance, [g/kg]	0.7 (0.6,0.9)	0.9 (0.7,1.0)	0.039

DR: dietary restriction; values presented as median (interquartile range). Δ-values represent changes of parameters between specified time points.

Despite using a computer-generated randomization system there was a significant difference in median serum creatinine at the timepoint of screening (DR group: 1.4(1.1,1.9) mg/dL, control group: 1.2(1.0,1.4) mg/dL; p = 0.009) and at pre-PCI baseline (DR group: 1.3(1.1,1.7) mg/dL, control group: 1.1(0.9,1.4) mg/dL; p = 0.004), which was also reflected in a difference in eGFR CKD-EPI (estimated glomerular filtration rate Chronic Kidney Disease Epidemiology Collaboration) before PCI: DR group: 50(34,62) mL/min, control group 62(50,79) ml/min/1.73m^2^; p = 0.002).

The total dose of administered contrast media was not different between the two groups (DR: 85(60,120) mL vs. control group: 90(73,128) mL, p = 0.329). With 6(3,9) in the DR group and 5(3,7.5) in the control group the median Mehran’s score predicting the risk on CI-AKI was statistically not different (p = 0.200), representing a low to moderate risk for CI-AKI in both groups.^26^ In 15 patients (37.5%) in the DR group and 18 patients (45%) of the control group percutaneous transluminal coronary angioplasty/additional stenting was performed. Coronary intervention-related parameters are summarized in Table 3.

**Table 3:** Percutaneous Angiography Data.

	DR group (n = 40)	control group (n = 40)	total (n = 80)	p
Duration of angiography [min], median (Q1–Q3)	56 (39,85)	4 (35,66)	50 (35,77)	0.179
Dosing of contrast agent [mL], median (Q1–Q3)	85 (60,120)	90 (73,127.5)	90 (60,125)	0.329
≤100 mL contrast agent, [n]	26 (66.7%)	24 (60.0%)	50 (62.5%)	0.644
>100 mL contrast agent, [n]	13 (33.3%)	16 (40.0%)	29 (36.3%)	0.485
PTCA/Stenting, [n] (%)	15 (37.5%)	18 (45.0%)	33 (82.5%)	0.650
1 vessel coronary artery disease, [n]	11 (27.5%)	4 (10.0%)	15 (18.8%)	0.045
2 vessel coronary artery disease, [n]	8 (20.0%)	10 (25.0%)	18 (22.5%)	0.592
3 vessel coronary artery disease, [n]	8 (20.0%)	13 (32.5%)	21 (26.3%)	0.204

DR: dietary restriction; PTCA: Percutaneous transluminal coronary angioplasty.

### Primary outcome

**Figure 2:**
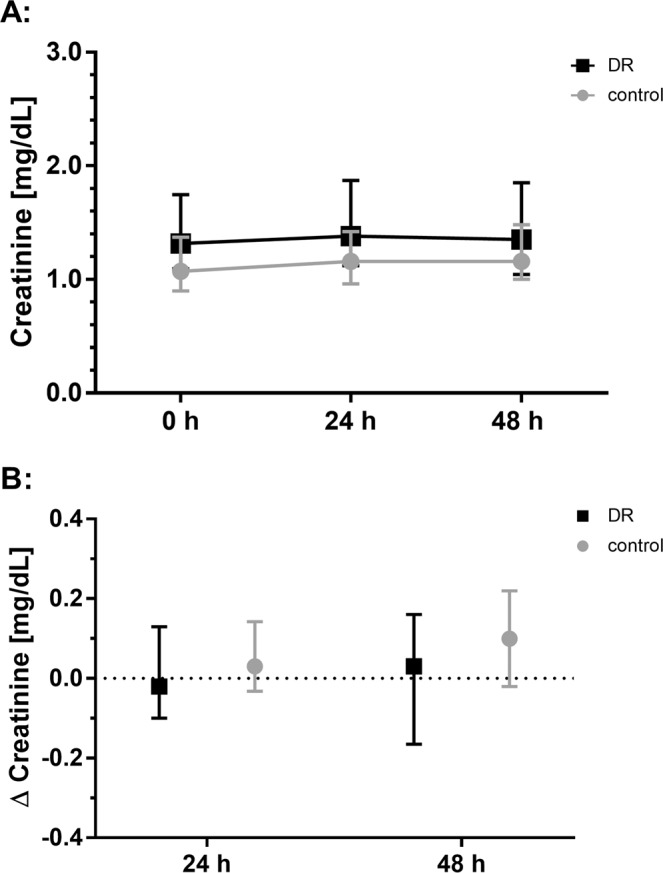
Intention-to-treat analysis: Evolution of serum creatinine (median, IQR) from pre-percutaneous coronary intervention (PCI) to 24 h and 48 h post PCI. (**A**) Development of serum creatinine from pre-PCI (0 h) baseline to 48 h post PCI. (**B**) Change of serum creatinine (Δ creatinine) from pre-PCI baseline to specified time points. DR: dietary restriction.

With a median Δ creatinine from pre-PCI baseline to 48 h post PCI of 0.03 (−0.15, 0.14) [mean ± SD: 0.06 ± 0.33] mg/dL for DR group vs. 0.09 (−0.03, 0.22) [mean ± SD: 0.10 ± 0.20] mg/dL for control group (p = 0.797), there was no difference in the primary endpoint (Fig. 2). Of note, although creatinine was significantly higher directly before PCI in the DR group compared to the control group, the inclusion of this parameter as a covariate in the analysis showed no significant influence on outcome (p = 0.947). Also, anthropometric and bioimpedance characteristics did not significantly affect the primary endpoint (Supplemental Table 2+3).

### Secondary outcomes

**Figure 3:**
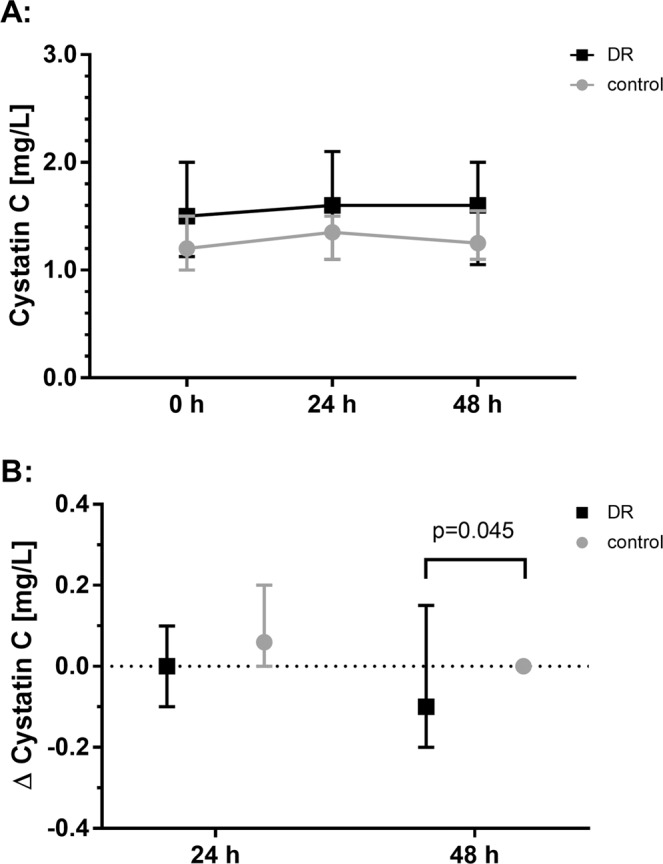
Intention-to-treat analysis: Evolution of serum cystatin C (median, IQR) from pre-percutaneous coronary intervention (PCI) to 24 h and 48 h post PCI (**A**) Development of median serum cystatin C from pre-PCI (0 h) baseline to 48 h post PCI. (**B**) Median values showing the change of serum cystatin C (Δ cystatin C) from pre-PCI baseline to specified time points. DR: dietary restriction.

There were no differences between the DR group and the control group with regard to the creatinine changes from pre-PCI baseline to 24 h. Also, the additionally assessed dynamics of the biochemical parameter urea, NGAL (only pre-PCI baseline to 24 h), CRP, WBC, CK and LDH did not show a difference between the groups from pre-PCI baseline to 24 h and 48 h (Table 4). Cystatin C slightly increased at 24 h in the control group, while there was no change in the DR group. Interestingly, a slight decrease in cystatin C at 48 h was observed in the DR group (48 h: CR: −0.10(−0.20, 0.10) mg/L, control: 0.00 (0.00, 0.14) mg/L; p = 0.045). For cystatin C evolution see Fig. 3.

**Table 4:** Secondary endpoints: biochemical parameters.

	DR group (n = 40)	control group (n = 40)	p
Cystatin C: d0 [mg/L]	1.5 (1.15,2.0)	1.2 (1.0,2.0)	<0.001
Δ Cystatin C: d0 to 24 h post PCI, [mg/L]	0.0 (−0.1,0.1)	0.1 (0.0,0.2)	0.077
Δ Cystatin C: d0 to 48 h post PCI, [mg/L]	−0.1 (−0.2,0.1)	0.0 (0.0,0.1)	0.045
NGAL in urine: d0, [µmol/L]	13.8 (10.0,26.3)	10 (10.0,15.8)	0.058
Δ NGAL: d0 to 24 h post PCI, [µmol/L]	2.5 (−0.1,27,2)	5.9 (0.0,13.0)	0.914
CK: d0, [U/L]	103 (67,121)	84 (62,114)	0.364
Δ CK: d0 to 24 h post PCI, [U/L]	−8 (−23,10)	−1 (−17,26)	0.265
Δ CK: d0 to 48 h post PCI, [U/L]	−3 (−20,20)	7 (−12,26)	0.278
CK-MB: d0, [U/L]	14 (12,18)	15 (13,16)	0.957
Δ CK-MB: d0 to 24 h post PCI, [U/L]	−1 (−4,1)	0 (−2,3)	0.119
Δ CK-MB: d0 to 48 h post PCI, [U/L]	−2 (−5,1)	−3 (−6,1)	0.323
LDH: d0, [U/L]	197 (183,244)	202 (182,232)	0.899
Δ LDH: d0 to 24 h post PCI, [ng/L]	10 (−22,34)	12 (−2,29)	0.513
Δ LDH: d0 to 48 h post PCI, [ng/L]	−2 (−23,31)	2 (−13,26)	1.000
CRP: d0, [mg/L]	3.0 (3.0,3.5)	3.0 (3.0,3.4)	0.995
Δ CRP: d0 to 24 h post PCI, [mg/L]	0.0 (0,0.7)	0.0 (0,0.6)	0.645
Δ CRP: d0 to 48 h post PCI, [mg/L]	0.8 (0.0,2.8	0.3 (0.0,2.4)	0.692
WBC: d0, [x1E9/L]	6.0 (5.0,7.7)	6.4 (45.5,7.9)	0.695
Δ WBC: d0 to 24 h post PCI, [x1E9/L]	0.93 (0.21,1.62)	1.25 (0.42,2.11)	0.316
Δ WBC: d0 to 48 h post PCI, [x1E9/L]	1.36 (0.68,1.70)	1.05 (0.22,1.81)	0.506

Parameters measured in serum unless otherwise indicated. 0 h indicates baseline value before PCI. Values presented as median (interquartile range). DR: dietary restriction; CK: creatine kinase; CK-MB: muscle-brain type creatine kinase; CRP: C-reactive protein; LDH: lactate dehydrogenase; NGAL: neutrophil gelatinase–associated lipocalin; WBC: white blood cell count. Δ-values represent changes of parameters from pre-PCI baseline (d0) to specified time points after PCI.

With 22.5% (DR) and 20.0% (control) the incidence of AKI defined according to the KDIGO criteria was similar (p = 0.602). When CI-AKI was defined as an increase in creatinine by >25% or 0.5 mg/dl within 48 h, the incidence was 17.5% and 20% in the DR group and the control group, respectively (p = 0.775) (Table 5). No death was reported during the course of the study and no patient received RRT. There was no difference in the number of hospitalizations in both groups (DR: n = 1, control: n = 1)

**Table 5:** Secondary endpoints: clinical parameters.

	DR group (n = 40)	control group (n = 40)	total (n = 80)	p
AKI, [n]	9 (22.5%)	8 (20%)	17 (21.3%)	0.602
KDIGO 1, [n]	8 (20%)	8 (20%)	16 (20.0%)	
KDIGO 2, [n]	0 (0.0%)	0 (0.0%)	0 (0.0%)	
KDIGO 3, [n]	1 (2.5%)	0 (0.0%)	1 (2.5%)	
CI-AKI, [n]	7 (17.5%)	8 (20.0%)	15 (18.8%)	0.775

DR: dietary restriction; AKI: acute kidney injury; KDIGO: Kidney Disease: Improving Global Outcomes; CI-AKI: Contrast induced acute kidney injury defined as an increase in serum creatinine by ≥0.5 mg/dL or by 25% from pre-PCI baseline within 48 hours.

### Subgroup analyses

**Figure 4:**
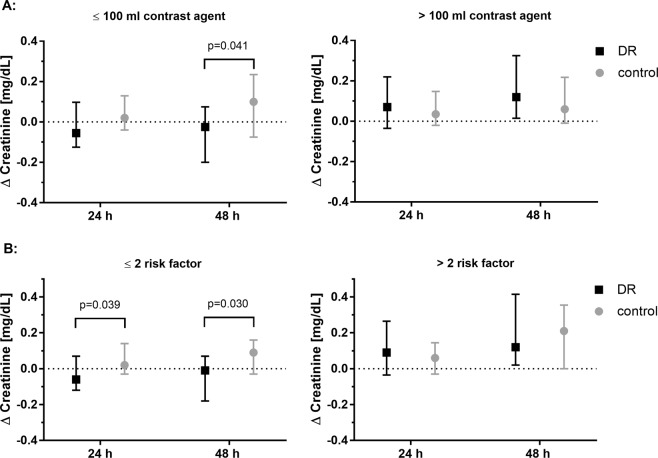
Subgroup analyses showing the median change of serum creatinine (Δ creatinine) from pre-PCI baseline to specified time points (**A**) subsets of patients with administration of ≤100 mL contrast agent (DR: n = 26, control: n = 24) and patients with administration of >100 mL contrast agent (DR n = 13, control: n = 16) (**B**) subsets of patients with ≤2 risk factors (DR: n = 27, control: n = 31) and >2 risk factors (DR: n = 13, control n = 9) for CI-AKI as defined by the inclusion criteria (age > 70 years, chronic kidney disease with a pre-existing serum creatinine above upper level of the normal range (i.e. >1.1 mg/dL in men, >0.9 mg/dL in women), diabetes mellitus, congestive heart failure NYHA 3–4 or reduced left ventricular ejection fraction (<50%) or peripheral vascular disease). DR: dietary restriction.

Subgroup analyses were performed for gender, BMI (BMI < 25 vs. BMI ≥ 25) and administered contrast agent volume (≤100 mL, >100 mL) as well as CI-AKI risk factors (≤ 2 vs. >2 risk factors as defined by the inclusion criteria). No between-group differences were seen with regard to gender and BMI. However, in the subset of patients that had received ≤100 mL of contrast agent, there was a significant difference in Δ creatinine between the two groups with a decrease in creatinine at 48 h in the DR group and a further increase in the control group (DR n = 26: −0.03(−0.20,0.08) mg/dL vs. control n = 24: 0.10(−0.08,0.24) mg/dL; p = 0.041). No difference in creatinine kinetics could be noted for patients that had received more than 100 mL contrast agent. Moreover, a significant beneficial impact of DR was also detected in the subgroup of patients with ≤2 risk factors for CKI-AKI (n = 27) with a decrease in creatinine of −0.06 (−0.12,0.07) mg/dL at 24 h and of −0.01 (−0.18,0.07) mg/dL at 48 h in the DR group vs. an increase of 0.02 (−0.03,0.14) mg/dL at 24 h and 0.09(−0.03,0.16) mg/dL at 48 h in the control group (24 h: p = 0.039; 48 h: p = 0.030), respectively. No differences regarding Δ creatinine were noted in patients with more than 2 risk factors (DR: n = 13, control: n = 9; 24 h: p = 0.647; 48 h: p = 0.744). Results of subgroup analyses are displayed in Fig. 4.

### Per-protocol analysis

As in the primary analysis no statistically significant differences were found with regard to the primary outcome parameter. However, per-protocol analysis confirmed the findings of the intention-to-treat analysis and revealed a difference in cystatin C increment in favor for the DR group 48 h after PCI (CR n = 30: –0.1(−0.2,0.1) mg/L, control n = 32: 0(0,0.14) mg/L; p = 0.018). Moreover, the subgroup analysis of the per-protocol population confirmed the finding, that only patients with ≤2 risk factors for CI-AKI showed a benefit of DR (n = 24) with a slight decrease in creatinine of −0.03(−0.18,0.10) mg/dL at 48 h, compared to an increase of 0.09(−0.04,0.18) mg/dL in the control group (n = 30) (p = 0.033).

Likewise, only the subset of patients that had received ≤100 ml of contrast agent experienced a decrease of creatinine in the DR group after 48 h (n = 24: −0.03(−0.2,0.09) mg/dL, whereas an increment was noted in the control group (n = 24: 0.1(−0.08,0.24) mg/dL, p  = 0.058).

### Safety and feasibility

Adverse events were assessed in the period starting with the first study-related procedure and ending with the last telephone contact at week 4 post-PCI. The diet was generally well tolerated. In 59.4% of documentations DR patients described their general condition during the diet as “rather good”, 36.9% reported a “fair” condition and only 3.8% of patients felt “rather not good”. In only 5.6% a sense of hunger was reported as “severe” while the majority of patients (66.3%) classified this sensation as “minor”.

One hospitalization due to progression of coronary artery disease was recorded in each group and classified as serious adverse event. Cardiac biomarkers (creatine kinase, muscle-brain type creatine-kinase) showed no differences in the DR group in comparison to the control group (Table 4). Likewise, DR had no negative impact on markers of inflammation (CRP, WBC) or cellular integrity (LDH), supporting the favorable safety profile of the intervention.

## Discussion

In this randomized controlled trial, 4-day DR aiming for a 40% reduction of calorie intake in patients at risk for CI-AKI showed no beneficial impact on the increase in serum creatinine at 48 h after PCI. Analysis of urinary NGAL – a marker of tubular injury – and incidence of AKI post-PCI was in line with this result. A small but significant protective effect was observed when cystatin C was used to monitor kidney function. Notably, DR proved to be effective in preventing CI-AKI (as mirrored by creatinine kinetics) in distinct subgroups of patients, i.e. those that received only a low contrast agent volume (≤100 mL) and those with a low- to intermediate-risk for CI-AKI prior to the intervention. This suggests that the applied DR regime provided only low-effective protection which was not appropriate to counteract a more severe insult. At last, our study clearly showed that short-term DR in patients undergoing PCI is feasible, well tolerated and safe.

Conducting a trial on CI-AKI one should also take into account that a controversial discussion about risk of CI-AKI as well as clinical relevance has emerged during the realization of this study. In a meta-analyses of 28 studies, involving 107,335 patients, Aycock *et al*. reported no difference in the rates of acute kidney injury, the receipt of renal replacement therapy and mortality in patients receiving contrast-enhanced CT versus those receiving non-contrast CT. Apart from the observational and largely retrospective nature of the analysis, which is also stated as an obvious limitation by the author, Aycock *et al*. quote, that this finding, which differs from the results of previous analyses by other authors, may be partially due to the fact that only patients undergoing CT scans were evaluated and patients undergoing other procedures were not included. Another important point is that “There is an inherent selection bias that comes from these studies as a result. Because none of the studies randomized patients to either contrast or non-contrast material, the type of CT scan ordered would have been prompted by indication for the scan or baseline renal function because of physician fear of postcontrast acute kidney injury”^27^

In another retrospective study, Wilhelm-Leen *et al*. examined the risk of contrast-associated nephropathy among patients hospitalized in the United States in 2009. 5,931,523 hospitalizations were included in the analysis, where Willhelm-Leen and colleagues found no difference for the risk for developing AKI in patients to whom radiocontrast was and was not administered. An important limitation is, that the administrative diagnosis of AKI exclusively served as the definition of AKI. One has to expect this to be less sensitive than clinical data, leading to an underestimation of overall incidence of AKI. Secondly, as the authors point out, the certainty in which the administration of contrast preceded the diagnosis of AKI could not be specified. Taken together, the recent data suggest, that the risk of CI-AKI might have been overestimated in the past, but even the authors admit that “the relationship between radiocontrast administration and AKI is highly confounded, unpredictable, and sometimes bidirectional”.^28^

In any case pathophysiology of CI-AKI in detail is still a matter of debate as are the different mechanisms that contribute to the phenomenon. Besides direct toxicity on renal tubular epithelial cells, reactive oxygen species formation and reduction of nitric oxide production as well as endothelial injury lead to hemodynamic alterations that promote renal medullary hypoxia.^29,30^ Most of the published clinical trials investigating preventive strategies have focused on one mechanism at a time and pharmacologic compounds that are usually administered directly before or together with the contrast media.^19^ However, such strategies are probably not appropriate to allow for profound protective adaptations on a cellular level and none of these approaches has reached the clinical setting. Recently, the principle of dietary preconditioning has gained large interest in this respect and might provide a distinct and innovative therapeutic approach.^3,10,31^ It has been known since more than 100 years that long-term reduction of food intake delays the aging process leading to an extension of healthy lifespan^32,33^ and various recent studies suggested that this effect is evolutionary conserved in a wide variety of species from yeast to mammals including primates.^34^ With the likely cause responsible for this phenomenon being a robust and longstanding enhancement of cellular stress resistance^35,36^ a short-term protection from acute stressors is achieved by brief periods of DR. With respect to the kidney, Mitchell *et al*. published a proof-of-concept study in a murine renal ischemia reperfusion injury model in which DR conducted for 2 to 4 weeks completely prevented death from AKI and dramatically ameliorated renal failure.^3^ Likewise, we showed recently that a 4-week 30%-CR strongly protects from toxic renal failure after cisplatinum application in mice.^35^

Despite a plethora of studies investigating dietary interventions with respect to weight control, only scarce data is available concerning the impact of DR protocols on organ protection in humans. In a large-scale clinical trial the administration of a calorie-restricted enteral feeding protocol in critically ill patients led to a significantly lower rate of renal replacement therapy.^37^ Furthermore, Jongbloed *et al*. confirmed the feasibility and safety of a preoperative calorie- and protein-restricted diet in healthy kidney donors as well as in morbidly obese patients undergoing bariatric surgery.^38^ In a preliminary analysis, this workgroup also observed a significantly lower incidence of acute renal graft rejection after transplantation in the DR group as compared to a non-dieting control group (personal communication by R.W.F. de Bruin, Erasmus MC, Rotterdam, The Netherlands). Recently, in a randomized controlled clinical trial evaluating the effects of a 7-day preoperative CR (60% of DEE) on renal function in patients undergoing cardiac surgery, a small albeit significant improvement on creatinine kinetics was revealed at 48 h after surgery and before discharge.^21^ Similar to the here reported trial, more pronounced beneficial effects were detected in distinct patient subgroups. Despite these promising data, the magnitude of the observed effects is considerably lower than it could have been expected from animal experiments. This may well be due to the fact that an effective clinical DR protocol has not been established yet. In contrast to rodent models, we simply do not know the optimal mode and extent of nutritional deprivation in order to achieve optimal protection in humans. Besides, reaching the same protection as in rodents may require longer-term exposure to DR in humans. Notwithstanding, with DR being a highly conserved and universally active hormetic principle, its exploitation might be beneficial for damage prevention in various settings – including those cases in which no alternative therapies exist. More recent data from cell culture and animal models regarding the restriction of specific components of the diet (e.g. protein reduction or restriction of sulfur-containing amino acids) show, that these regimens may activate the underlying cellular mechanisms in a targeted and more efficient fashion.^39^ Further research is therefore needed in order to refine DR protocols with respect to timing and formulation. In this context, the results of the study at hand as well as our previous work in cardiopulmonary bypass surgery provide a good basis showing – albeit modest – first positive effects in CI-AKI associated with a good safety profile and feasibility.

This study has several limitations, some of which derive from its design and the principle problem of adherence in diet studies. Blinding was not feasible given the fact that a reduction of calorie intake would readily be noticed by study participants. The widely accepted Mifflin-St. Jeor equation that was used in this study to calculate DEE, as any alternative published equation, might not be applicable in the given population. In addition, concealed adherence problems in the DR group and unintended dieting in the control group might have distorted the results as it has been observed in primate trials.^40^ In general, a precise surveillance of food intake and therefore calorie administration especially in the control group in our study was hindered by the ambulatory trial design and the difference between the modes of diet (formula diet vs. regular food) might represent an influential factor. The use of a formula diet at 100% of calculated DEE could serve as a control in future studies, although ingesting the large amount of formula diet to meet calorie requirements may be challenging.

In addition, the calculation of protein intake based on the urinary urea nitrogen appearance potentially leads to an overestimation of protein intake due to activation of nitrogen sparing responses. Calculation of the protein intake of the DR group from the amount of the ingested formula diet instead would suggest a reduction to approximately 45.5 g/day.

Eventually, although the significantly profound weight-loss in the DR group in addition to the – probably even still overestimated – reduced protein intake indicates a true dietary effect, the aimed-for between-group difference in nutritional intake of 40% was probably not achieved. The fact that the control group might have consumed less calories than 100% of their DEE contributed to this fact. Thus, the true effect of DR might be underestimated

Finally, despite the implementation of risk factors for CI-AKI in the inclusion criteria targeted for enrollment of patients at risk for CI-AKI, the protocol-inherent necessity of a time frame of 4 days before PCI consequently excluded high-risk patients (e.g. cardiogenic shock, intra-aortic balloon pump, etc.), which are usually subject to prompt PCI. Consequently, increase of creatinine lagged behind the assumed standardized effect, and calculated group size in this trial was presumably too small to obtain an appreciation of the true potential of DR.

## Conclusion

The results from this randomized controlled trial show that a 4-day 40%-DR does not prevent CI-AKI at 48 h after PCI. However, analysis of distinct subgroups detected discrete signals of renal protection. This finding together with the good safety and feasibility profile warrants further studies in order to appreciate the true protective effect of dietary interventions.

## Supplementary information

Supplemental material.

## Data Availability

Study protocol is provided as Supplemental material. Data described in the manuscript, code book, and analytic code will be made available upon request.
