# Supplementary material for: Dietary restriction for prevention of contrast-induced acute kidney injury in patients undergoing percutaneous coronary angiography: a randomized controlled trial
Source: Sci Rep. 2020 Mar 23;10:5202. doi: 10.1038/s41598-020-61895-2 (PMC7089976; doi:10.1038/s41598-020-61895-2)
Supplement: Supplementary file 1 — Supplemental material. [file 41598_2020_61895_MOESM1_ESM.docx]

**Supplemental material**

**Dietary restriction for prevention of contrast-induced acute kidney injury in patients undergoing percutaneous coronary angiography: a randomized controlled trial**

Franziska Grundmann*^1^, Roman-Ulrich Müller^1,2,5^, Karla Johanna Ruth Hoyer-Allo^1,2^,
Martin Richard Späth^1,2^, Eva Passmann^1^, Ingrid Becker^3^, Roman Pfister^4^,
Stephan Baldus^4^, Thomas Benzing^1,2,5^, and Volker Burst^1^

*^1^Department II of Internal Medicine and Center for Molecular Medicine Cologne, University of Cologne, Faculty of Medicine and University Hospital Cologne, Cologne, Germany*

*^2^CECAD, University of Cologne, Faculty of Medicine and University Hospital Cologne, Cologne, Germany*

*^3^Institute of Medical Statistics and Computational Biology, University of Cologne, Cologne, Germany*

*^4^Department III of Internal Medicine, Heart Center, University Hospital of Cologne, Cologne, Germany*

*^5^Systems Biology of Ageing Cologne, University of Cologne, Germany*

**Address correspondence to:**

Franziska Grundmann, Department II of Internal Medicine, University of Cologne, Kerpener Str. 62, 50933 Cologne, Germany, Email: franziska.grundmann@uk-koeln.de, Phone: +49 221 478 4480

**Supplemental Table 1:** Bioimpedance values at screening and day -1 (hospital admittance day)

|  | **DR group** | **control group** | **p** |
| --- | --- | --- | --- |
| Total body water at screening, [kg] | 44.1(35.8,48.4) | 43.7(37.3,48.2) | 0.858 |
| Total body water at day -1, [kg] | 42.3(37.0,44.7) | 43.0(35.7,49.2) | 0.331 |
| Δ body water: screening to day -1, [kg] | -2.1(-4.1,-1.0) | -1.1(-1.6,0.6) | 0.002 |
| Total body water [kg] / weight [kg] at day -1 | 0.50(0.48,0.58) | 0.52(0.48,0.56) | 0.735 |
| Total body impedance at screening, [Ω] | 506(476,592) | 556(485,617) | 0.347 |
| Total body impedance at day -1, [Ω] | 552(527,648) | 538(486,606) | 0.335 |
| Δ total body impedance: screening to day -1, [Ω] | 59 (28,83) | 17(-12,44) | <0.001 |
| Total body impedance at day -1/height, [Ω/m] | 322(298,372) | 308(284,359) | 0.331 |

DR: dietary restriction; values presented as median (interquartile range). Δ-values represent changes of parameters between specified time points.

**Supplemental Table 2:** Influence of bioimpedance values on primary endpoint

| **Covariate** | **Significance testing (p) of influence of the covariate on primary endpoint** | **Significance testing (p) of model** |
| --- | --- | --- |
| Total body water at day -1, [kg] | 0.969 | 0.977 |
| Total body water [kg] / weight [kg] at day -1 | 0.789 | 0.965 |
| Δ total body impedance: screening to day -1, [Ω] | 0.366 | 0.664 |
| Total body impedance at day -1/height, [Ω/m] | 0.906 | 0.779 |

DR: dietary restriction; p values acquired by ANCOVA, Δ-values represent changes of parameters between specified time points.

**Supplemental Table 3:** Correlation of bioimpedance values with primary endpoint in complete patient cohort.

| **Bioimpedance values** | **Spearman Correlation Coefficient** | **Significance testing (p)** |
| --- | --- | --- |
| Total body water at day -1, [kg] | 0.03 | 0.784 |
| Total body water [kg] / weight [kg] at day -1 | 0.02 | 0.860 |
| Height^2^/total body impedance at day -1, [m^2^/Ω] | 0.04 | 0.766 |
| Total body impedance at day -1, [Ω] | -0.05 | 0.736 |
| Δ total body impedance: screening to day -1, [Ω] | 0.09 | 0.547 |
| Total body impedance at day -1/height, [Ω/m] | -0.05 | 0.699 |

Δ-values represent changes of parameters between specified time points.
